# St. George's Hospital

**Published:** 1913-12-13

**Authors:** 


### THE EDITOR'S LETTER-BOX.

### St. George's Hospital.

*To the Editor of* The Hospital.

Sir,—What your correspondent said in your last issue about St. George's Hospital is true enough. Up to the time of Mr. Todd's retirement it had a prosperous career, its revenue increased year by year, and though it was then, as it is now, one of the most expensively worked hospitals in London, it paid its way and was able to put something by. Then came a sort of general upheaval. The honorary medical staff became divided against itself and at issue with the governors, changes of secretaries, or what stood for secretaries, added to the confusion, and those who made themselves responsible for getting in the income proved their incapacity to do so. This is a pitiful state of affairs, for the hospital has done a great work in the past; it is always in evidence, and is situated in the most wealthy neighbourhood in the Metropolis. It is time that the governors appointed someone who knows the difficulties that beset a hospital, who understands the somewhat complex etiquette of the medical profession, and has had experience in dealing with a large body of astute and able women. This is the only course that will regain the confidence of the public and restore St. George's to its old position in the hospital world.—I am, Sir, etc., M.

The Primrose Club, Park Place, St. James's, S.W.,
December 6, 1913.

